# Type I Interferons in Newborns—Neurotoxicity versus Antiviral Defense

**DOI:** 10.1128/mBio.00639-16

**Published:** 2016-05-17

**Authors:** Dusan Bogunovic

**Affiliations:** Department of Microbiology, Department of Pediatrics, The Mindich Child Health and Development Institute, Icahn School of Medicine at Mount Sinai, New York, New York, USA

## Abstract

In most children and adults, primary infection with herpes simplex virus 1 (HSV-1) is asymptomatic. However, very rarely (incidence of 1 in 1,000,000), it can cause herpes simplex encephalitis (HSE). HSE also occurs in infants but with a much starker incidence of one in three. This age difference in susceptibility to HSV-1-caused HSE is not well understood. In a recent article in *mBio*, authors have identified the choroid plexus as the anatomical site of robust HSV-1 replication in the brain. They point to low levels of type I interferon (IFN) receptor as causal of the lack of HSV-1 replication control in neonates, in contrast to adults. Here, I discuss these findings in the context of human genetic evidence. I point to the balancing act of type I IFN acting as a neurotoxin and an antiviral agent, an evolutionary choice of a lesser evil.

## COMMENTARY

Primary infection with herpes simplex virus 1 (HSV-1) mostly results in asymptomatic host responses. If symptoms do occur, they are generally limited to what is commonly referred to as “cold sores.” However, HSV-1 may, in very rare cases, cause disseminated or brain-specific disease in children and adults. HSV-1 has a seroprevalence of about 60 to 85% of the population in most countries worldwide, while herpes simplex encephalitis (HSE) has an estimated incidence of about 1 in 1,000,000 individuals. A genetic contribution to the development of HSE in children (not neonates) and adults following primary infection has been identified, showing the Toll-like receptor 3 (TLR3) pathway to be nonredundant in the control of HSV-1, particularly in neurons and oligodendrocytes. HSE is generally sporadic rather than familial, but mutations of *TLR3*, *UNC93B1*, *TRIF*, *TRAF3*, *TBK1*, and *IRF3* have been identified as causal of HSE, and the likelihood of developing HSE is thought to be about 100 times higher than that in the general population for carriers of severely hypomorphic or null *TLR3* alleles (1). A broader infectious phenotype including HSE has been reported in human deficiencies affecting the type I interferon (IFN) pathway in patients with *STAT1* and *NEMO* germ line mutations (1).

HSE has an overtly higher incidence in neonates (up to the age of 12 weeks) than in older children and adults. Most HSE cases occur within 6 weeks of birth. In this particular population, it is estimated that infection leads to HSE development in one in three cases. This susceptibility to infection wanes to adult-like levels within a few weeks, highlighting the rapid immunological changes occurring in neonates. Precise physiological evidence of a difference between primary HSV-1 infection in neonates and adults remains incomplete, although a recent article in *mBio* provides important new insights (2).

The basis of susceptibility to HSV-1 in the first 12 weeks of life is probably not genetic, given that most infected newborns present symptoms, which are severe in two-thirds, with one-third developing HSE. Neonatal HSV-1 infection is associated with very high levels of morbidity and mortality, despite treatment with acyclovir, which some argue remains underused in this population of patients. In a recent article in *mBio*, Wilcox et al. (2) note important differences between neonatal and adult HSV-1 infections. The authors have identified the choroid plexus in newborn mice and in postmortem neonatal human brains as the anatomical site of robust HSV-1 replication. By determining the levels of a number of innate immune effectors in the choroid plexus in neonates and adults, they also identified significantly lower levels of the IFN receptor (IFNAR) in the neonate choroid plexus as a cause of susceptibility to HSV-1, providing an increased understanding of HSE pathogenesis in newborns.

Another important and even broader question arising from these findings is that of evolutionary advantage. What benefit does the neonate derive from having lower IFNAR levels, which may place it at risk of death or life-long severe neurological sequelae if infected with HSV-1 in the first 3 months of life? According to Occam’s razor, the simplest answer is usually correct. The simplest answer here is that having levels of IFNAR during intrauterine development and the first 12 weeks of life that are similar to those of adults is more detrimental to perinatal health than the likelihood of infection with HSV-1. There is growing evidence that suggests that this simplest answer is, indeed, correct.

Let us examine two lines of evidence from human genetics. The first comes from individuals with too much IFN activity during perinatal development, mimicking an overabundance of IFNAR and its binding to IFN. Type I interferonopathies are disorders that prominently feature the dysregulation/persistence of the IFN signal. The genetic dissection of a particular type I interferonopathy, Aicardi-Goutières syndrome, initially described as an early-onset progressive brain disease, identified mutations of the *TREX1*, *RNASEH2A*, *RNASEH2B*, *RNASEH2*C, *SAMHD1*, *ADAR*, and *MDA5* genes as causal*.* Other genetic causes of type I interferonopathies have been identified. Patients with mutations of *ISG15*, *USP18*, *RIG-I*, *STING*, *PSMB8*, and *ACP5* also display systemic features of IFN dysregulation (3, 4). Severity and onset times differ between patients with the above-mentioned type I interferonopathies, even if they carry the same mutations, but early-onset neurologic and autoinflammatory symptoms are particularly common in all type I interferonopathies. In essence, these experiments of nature tell us that too much IFN too early is neurotoxic. There is therefore an evolutionary advantage to taming the response to IFN during development *in utero* and very early in life.

The other line of evidence concerns the situation in which the response to IFN is very weak or absent. The first five humans with a deficiency in STAT2, an essential component of IFN signaling, were reported in 2013. STAT2 deficiency results in an IFN signaling deficiency, although some noncanonical signaling may occur. Of note, although viral illness at times was more severe than usually observed (reaction to measles, mumps, and rubella [MMR] vaccine, in particular), these patients displayed normal control of Epstein-Barr virus infection, varicella zoster virus infection, cytomegalovirus infection, non-life-threatening HSV-1 gingivitis, and some respiratory infections. The one observed case of severe disseminated infection leading to death occurred after an unknown viral infection in an infant, suggesting that STAT2 may play an essential but narrow role in early childhood (5). In 2015, a genetic deficiency of IFNAR itself (a homozygous deletion in IFNAR2, a subunit of the IFNAR complex) was discovered in a human patient (6). The affected child had normal intrauterine and neonatal development. During the entire first year of this patient’s life, no abnormalities associated with common viral infections were observed. The deficiency, fatal in the end, was revealed after MMR vaccination (the patient was negative for HSV-1 and HSV-2, and the newborn sibling who also carries the mutation is doing well). These findings on IFNAR deficiency also suggest that IFN is essential, but only for a fairly narrow activity spectrum of infections.

Overall, a comparison of type I IFN pathway over- and underactivity in these human deficiencies teaches us two important physiological lessons. The first is that IFN signaling is not required for intrauterine, neonatal, or early childhood neurodevelopment. In contrast, overactive IFN signaling is detrimental to neurodevelopment. When the overactivity is mild enough, it can lead to a gain-of-function antiviral phenotype, as documented for ISG15 deficiency (7). The second key finding is that an absence of IFN signaling can be fatal, as after MMR vaccination in this case, but that most affected children manage to control most common childhood infections without complications. There is thus a tradeoff concerning IFN levels: neurotoxicity versus antiviral activity.

In conclusion, the evidence suggests that natural selection has chosen the best balance for us but that this balance is not perfect. Lower IFNAR (and IFN signaling) levels may protect us against the neurotoxic effects of IFN during development. After birth, as we gradually lose our maternal immune protection and become less sensitive to the effects of IFN on neurodevelopment, our responsiveness to IFN increases, enabling us to control viral infections. In neonates, HSV appears to take advantage of this gap in what is otherwise a beneficial evolutionary development. Much more work is needed to profoundly understand HSV-1 infection in newborns and to develop effective treatments for neonatal HSE.
